# Environmental Enteric Dysfunction: A Case Definition for Intervention Trials

**DOI:** 10.4269/ajtmh.17-0183

**Published:** 2017-09-25

**Authors:** Donna M. Denno, Phillip I. Tarr, James P. Nataro

**Affiliations:** 1 Department of Pediatrics, University of Washington, Seattle, Washington;; 2 Department of Global Health, University of Washington, Seattle, Washington;; 3 Department of Pediatrics, Washington University in St. Louis School of Medicine, St. Louis, Missouri;; 4 Department of Pediatrics, University of Virginia School of Medicine, Charlottesville, Virginia

Environmental Enteric Dysfunction (EED) was first described as a malabsorption syndrome with small intestinal villous flattening among healthy adults in Thailand and other low-resource settings.^1–4^ A similar entity was described in Peace Corps volunteers in South Asia.^5,6^ Several years later, resolution of histopathology and malabsorption after repatriation or immigration to North America was demonstrated.^7,8^

Ensuing studies described that a similar entity, also manifested by abnormal histopathology, permeability defects, inflammation, and impaired nutrient absorption, is widespread among impoverished children in settings with poor sanitation and hygiene.^9–14^ Recent data corroborate these findings, and associate increased gut permeability and inflammation with ponderal, and especially linear, growth faltering in children living in low-income countries.^15–19^ Such growth deficits have been associated with poor outcomes such as cognitive delay and chronic noncommunicable diseases in adulthood.^20–22^ Although the precise cause(s) of EED remain(s) elusive, exposure to fecally contaminated environments, specific microbes, and/or a paucity of beneficial intestinal bacteria are candidate precipitants.^19,23^

Recent and ongoing studies are assessing relationships between intestinal dysfunction and suboptimal growth in childhood. The Malnutrition and Enteric Diseases (MAL-ED) Network demonstrated that intestinal permeability defects were common among young children in Asia, Africa, and Latin America.^24^ The EED Biomarkers Initiative Consortium is identifying and validating candidate biomarkers and increasing our understanding of EED pathophysiology.^25^ Water, sanitation and hygiene (WaSH) trials are testing effects of environmental interventions on EED prevalence.^26,27^

However, even if WaSH efficacy trials demonstrate the ability of these interventions to reduce EED burden, this entity is unlikely to be eliminated soon, as efforts to implement WaSH at high coverage have been inadequate. For example, at least 1.8 billion people worldwide are estimated to drink fecally contaminated water, and only 68% have access to “improved” sanitation facilities, as defined by the United Nations Joint Monitoring Program.^28^ Also, the capacity to implement hygiene practices is constrained because of insufficient access to water of any quality. Hence, even if WaSH can prevent EED, many children will continue to suffer from EED and its consequences, and substantial interest exists in identifying and trialing therapeutics.^29^

Given its obscure etiology, largely sub-clinical nature, and no clear role for a single biomarker to identify a case, it is currently impossible to diagnose a specific child with EED. This uncertainty has impeded the design of clinical trials for this important entity. Recently, Keusch et al.^30^ introduced a paradigm shift toward functional gastrointestinal deficit as a defining feature of EED, but acknowledged the challenges in formulating a well-circumscribed, clinically and epidemiologically useful, durable definition. Thus, a formal case definition of EED will constitute a significant advance in the study of therapeutic interventions.

Therapeutics trials necessarily use more cautious case definitions than those used in prevention studies, which generally recruit community-based asymptomatic/presymptomatic individuals and test interventions with little or no risk (e.g., WaSH interventions). Therapeutics pose risks that are at least hypothetically greater than those tested in prevention studies. An EED case definition within the context of therapeutic trials, should, therefore be sufficiently specific to exclude individuals unlikely to have EED, to minimize potential harm, and maximize possible benefit. Such specificity also reduces potential misclassification and false ascertainment of lack of efficacy, as the likelihood of identifying a true effect diminishes because of misclassification (i.e., type 2 error).^31^ In other words, if children do not “respond” because they do not have the condition, a null finding is more likely, thereby leading to potentially dismissing a treatment that might work.

To advance the EED field, we now propose a definition construct analogous to the Jones criteria for acute rheumatic fever, intended for therapeutic trials, and which can be modified as new data emerge. In building this proposal, we recognize that EED is, except for poor growth, rarely accompanied by clinical signs or symptoms, so our criteria highly depend on laboratory assessment. Accordingly, our definition is based on three domains, which, taken together, indicate an acquired intestinal inflammatory disorder with substantial clinical impact. Domain 1 includes age, presence of linear growth failure, negative celiac disease testing; domain 2 includes gut histopathology consistent with EED or at least two intestinal deficits assessed by less invasive biomarkers; domain 3 consists of biomarkers nonspecific to enteric dysfunction, but representing consequences of EED. We discuss a “sliding scale” application of the proposed criteria based on the adverse event potential of the therapy. Finally, we propose a definition of response to intervention.

## DOMAIN 1. INCLUSION OF EACH OF THE FOLLOWING ELEMENTS

### Age: 2–36 months.

EED is an acquired condition caused by an environmental exposure, and attempts to reverse linear growth failure become increasingly futile beyond 36 months,^32^ thereby justifying the lower and upper ages for applying the definition. Extension beyond this span can be considered, especially if linear growth is not being assessed as a primary outcome.

### Clinically consequential disease manifest as linear growth faltering.

Insufficient change in height-for-age *Z* score (HAZ) is preferred over point assessments, e.g., stunting (HAZ < −2) which has often been used as an outcome in trials, but such a categorical endpoint is problematic.^33^ Stunting is better used as an indicator of chronic malnutrition population prevalence than as a metric of individual growth.

We recommend that the case definition include, in the context of a child receiving routinely recommended nutrition for age^34^: 1) a 10% decline in HAZ over ≥ 90 days when the starting HAZ is < −1 to > −3; or 2) no improvement in HAZ in a child who is already severely stunted (HAZ < −3).

Linear growth deficits often co-exist with ponderal growth insufficiency. Any child with acute malnutrition, regardless of linear growth status, should be nutritionally rehabilitated per guidelines.^35–37^ We acknowledge that there are many causes of malnutrition other than EED, including food insecurity. Linear growth responsive solely to standard nutritional rehabilitation aimed at correcting wasting should not be assumed to be EED. However, children meeting the case definition of EED who have concurrent acute malnutrition unresponsive to relevant recommended management may represent priority candidates for EED therapeutics.

### Negative screening for celiac disease.

Appropriate testing should exclude celiac disease as a cause of enteric dysfunction and linear growth shortfall.^38^ This criterion could be adjusted based on HLA DQ2/DQ8 risk allele prevalence in the target populations.

## DOMAIN 2

We suggest that at least two biomarker categories specific to gut dysfunction be abnormal if histopathology is unavailable. The biomarker categories encompass several pathologic mechanisms underlying EED.^39^ The exact biomarkers within the categories can be adapted as new data emerge.

### Histopathology.

Obtaining biopsies is often infeasible, but they are considered the “gold standard” test. Ongoing EED histopathology studies will likely further inform the field. For now, traditional measures of gut histopathology can be used, such as:

- Normal villus height:crypt depth ratios in children are as low as 2:1, as noted in attempts to define cut-points for these values in celiac disease. We propose that ratios less than this value be considered to reflect a process consistent with EED.^40^
- Intraepithelial lymphocyte (IEL) densities < 20 or < 25 per 100 enterocytes have been recently suggested, whereas older literature proposed higher cut-points based on Crosby capsule-obtained specimens which sampled more distally into the jejunum which has higher IEL densities.^41–44^

### Intestinal permeability biomarker.

Dual sugar permeability testing involves ingestion of a large sugar molecule (e.g., lactulose [L]) which should not cross a healthy nonpermeable gut, and a smaller sugar (e.g., mannitol [M] or rhamnose [R]) which more readily traverses the mucosa. There have been few reliable normative values for these tests among children living in healthy environments in low- and middle-income countries.^45^ However, Kosek et al.^24^ recently published normative sex- and age-based *Z* scores for L:M as well as for percent excretion of lactulose and mannitol, based on MAL-ED data from Brazilian 3 to 15-month olds with healthy growth and low diarrhea incidence. By 15 months of age, ∼15–45% of children at all but one of the other MAL-ED sites (Bangladesh) had L:M values above the Brazil 90th percentiles with ratios ranging from 0.2 to 0.7, depending on age and sex. A cut-point for L:R of > 0.7 has been suggested to indicate increased permeability based on data from 2 to 13-month olds in the United States.^46^ We endorse these cut-points—unless and until further normative data suggest otherwise. In addition, we recommend reporting the percent excretion of the individual sugars, as emerging data suggest heterogeneity in mechanisms driving ratio results (i.e., absorption versus permeability).^24^

We recognize the technical challenges associated with dual sugar permeability testing.^45,47^ However, increased permeability, and resulting translocation are believed to be hallmarks of EED. It may be possible to use other markers, such as fecal alpha-1 antitrypsin^17,48^; nonetheless, we believe that dual sugar permeability testing, its technical challenges notwithstanding, has value as a functional, as opposed to static, assessment of gut barrier disruption.

### Translocation biomarker.

We cannot yet recommend a specific marker of translocation, however, we endorse incorporation of such measures according to emerging data.

### Intestinal inflammation biomarker.

Data suggest that fecal myeloperoxidase (MPO) is a useful test of gut inflammation and that it predicts linear growth deficits.^17,48–50^ For example, in the first MAL-ED publication on MPO by Kosek et al.,^17^ concentrations > 75th percentile predicted 0.293 HAZ loss (versus. stools concentrations ≤ 25th percentile). However, absolute cut-points for this assay are difficult to endorse at this time. While a value of 2,000 ng/mL has been used, this cut-point is derived from specimens from adults living in a high-income setting,^51^ and Kosek et al.^17^ demonstrated mean MPO concentrations from across MAL-ED sites that were several-fold greater. Subsequent MAL-ED analyses also demonstrated high prevalences of MPO concentrations in excess of 2,000 ng/mL, but these studies reported lower central tendency and upper range concentrations than did Kosek et al.,^17^ and had considerable geographic and age variability.^47–49,52^ Currently, we propose a cut-point of 2,000 ng/mL, but it is likely that emerging data will inform more useful normative values.

### Reduced absorption biomarker.

Zinc absorption assessed by stable isotope can be complex to measure, but might offer insight into intestinal absorptive function. Fractional absorption of zinc levels should be considered in relation to the amount of radiolabeled zinc ingested.^53^

### Intestinal cellular generation or repair.

Stool regenerating gene 1 (REG1) proteins may be involved in intestinal cellular growth, repair and regeneration and fecal REG1B has been associated with lower linear growth in Bangladeshi and Peruvian children.^54^

### Gut microbial populations or specific components biomarker.

We cannot yet recommend pursuit of a particular organism or microbiome profile. The microbiome maturation index or the detection of specific organisms or combination of organisms might eventually serve as a diagnostic feature.^55^

## DOMAIN 3. POTENTIAL SUPPORTING CRITERIA

Gut inflammation and translocation of microbes or their products can cause immune activation and systemic inflammation. However, other non-intestinal processes can also lead to immune activation and systemic inflammation. Hence, these markers should not be used in the absence of an intestine-specific indicator and investigators may wish to exclude subjects with intercurrent illness. Examples of systemic inflammatory markers are:^47,56^

- ESR > 20 mm/hour
- CRP > 5 mg/L
- WBC > 17 × 103/µLAGP > 1 g/L
- Ferritin, although levels depend on iron status as well as inflammation.

Markers of downstream metabolic effects can also be considered within this domain guiding by emerging data. For example, tryptophan conversion to kynurenine by indoleamine 2,3-dioxygenase 1 is augmented by immune activation or inflammation. Elevated tryptophan levels haven been associated with cytokines, CRP and AGP as well as decreased linear growth in children from Peru and Tanzania.^57^

## “SLIDING SCALE” APPLICATION OF DEFINITION

Staged approaches can also be taken based on expected intervention-related potential adverse events. Clinical manifestations will not likely be helpful as EED is largely asymptomatic. Investigators can titrate subject selection by relying on markers indicative of more severe pathophysiology, such as systemic inflammation, or using more extreme EED marker cut-points if an intervention has a higher adverse effect potential. In addition, therapeutic trial investigators may also wish to identify subsets of children based on pathophysiologic mechanisms related to the therapeutic target to be tested (e.g., individuals with markers of intestinal and/or systemic inflammation if trialing anti-inflammatories).

## DEFINITIONS OF RESPONSE TO TREATMENT

Responses can be grouped into two categories: as measures of 1) improved enteric function (i.e., less severe EED) or 2) improved linear growth. Whereas longer-term consequences such as cognitive development are of ultimate interest, studies of durations sufficient to assess such outcomes are unusual and identification of growth benefit in a shorter timeframe is a reasonable surrogate. An interval of at least 3 months is generally needed to determine if an intervention improves linear growth.^33^ We recommend coupling markers of inflammation and enteric function with linear growth changes as a robust and practical approach.

## CONCLUSION

Our framework should be considered to be a work in progress, able to accommodate new components as relevant data emerge. We acknowledge the embryonic state of the science and welcome modifications to our framework with advances in the field. In addition, it is possible that EED represents a spectrum of abnormalities, and future iterations of this definition might attempt to further capture these variations. Further understanding of the pathophysiology and consequences of EED is needed before applying a multipurpose definition for population surveillance, screening, diagnosis, and prevention and treatment research. Indeed, single definitions are possible for few public health problems. As new data emerge, we hope that case definitions for use in these contexts can also be developed. In the interim, we offer a definition for use in therapeutic trials.

In summary, an EED study case definition will enable treatment trials to proceed. Adherence to such a definition will improve studies’ abilities to identify a useful therapeutic, reduce risk from interventions among children who do not have EED, and allow interstudy comparisons of effect sizes and other outcomes. We anticipate many advancements in the EED field, and the definition can be updated in response. We look forward to scientific developments that will further refine our proposed definition.
